# Short-term outcomes of curved periacetabular osteotomy and factors influencing patient dissatisfaction

**DOI:** 10.1093/jhps/hnac054

**Published:** 2023-01-25

**Authors:** Masahiro Suzuki, Koichi Kinoshita, Tetsuya Sakamoto, Hajime Seo, Ichiro Yoshimura, Takuaki Yamamoto

**Affiliations:** Department of Orthopaedic Surgery, Fukuoka University Faculty of Medicine, 7-45-1 Nanakuma, Jonan-ku, Fukuoka 814-0180, Japan; Department of Orthopaedic Surgery, Fukuoka University Faculty of Medicine, 7-45-1 Nanakuma, Jonan-ku, Fukuoka 814-0180, Japan; Department of Orthopaedic Surgery, Fukuoka University Faculty of Medicine, 7-45-1 Nanakuma, Jonan-ku, Fukuoka 814-0180, Japan; Department of Orthopaedic Surgery, Fukuoka University Faculty of Medicine, 7-45-1 Nanakuma, Jonan-ku, Fukuoka 814-0180, Japan; Department of Orthopaedic Surgery, Fukuoka University Faculty of Medicine, 7-45-1 Nanakuma, Jonan-ku, Fukuoka 814-0180, Japan; Department of Orthopaedic Surgery, Fukuoka University Faculty of Medicine, 7-45-1 Nanakuma, Jonan-ku, Fukuoka 814-0180, Japan

## Abstract

This study aimed to evaluate clinical outcomes based on patient-reported outcome measures and to analyze factors influencing patient dissatisfaction at 1 year after curved periacetabular osteotomy (CPO). This retrospective review involved 98 hips of 98 consecutive patients with symptomatic acetabular dysplasia who underwent CPO from March 2016 to June 2020. The clinical outcomes were evaluated based on the Japanese Orthopaedic Association Hip Disease Evaluation Questionnaire, the Medical Outcomes Study 36-Item Short-Form Health Survey and the Harris Hip Score. Patients were divided into satisfied and unsatisfied groups according to the 85th percentile cut-off on the postoperative visual analog scale (VAS) for dissatisfaction. A multiple logistic regression analysis was performed to investigate the factors impacting patient dissatisfaction after CPO; the explanatory variables were age, body mass index, postoperative Tönnis grade of ≥2, postoperative VAS score for pain, lateral femoral cutaneous nerve (LFCN) injury and radiographic complications (non-union of the pubis, non-union of the ischium after posterior column fracture and ischial ramus stress fracture). The 85th percentile of the postoperative VAS score for dissatisfaction was 60 mm. The unsatisfied group comprised 15 patients at 1 year after CPO. The multiple logistic regression analysis results showed that the postoperative VAS scores for pain [odds ratio (OR), 1.064; 95% confidence interval (CI), 1.026–1.104; *P* = 0.001] and LFCN injury (OR, 6.775; 95% CI, 1.308–33.256; *P* = 0.018) were associated with postoperative dissatisfaction. LFCN injury and the postoperative VAS score for pain independently impacted postoperative dissatisfaction at 1 year after CPO.

## INTRODUCTION

Acetabular dysplasia of the hip is a cause of hip osteoarthritis [1]. Periacetabular osteotomy (PAO) has been widely performed for symptomatic acetabular dysplasia of the hip [2]. Good clinical outcomes after PAO have been reported [3–6], mostly using evaluation methods based on clinician-reported outcome measures. However, evaluation by physicians is influenced by their subjectivity and does not necessarily match patients’ perceptions.

Patient satisfaction and patient’s quality of life have been the focus of more recent investigations, and patient-reported outcome measures (PROMs) have been used to indicate treatment outcomes. Increasing numbers of reports are using PROMs, and good results have been reported after PAO [7–10]. A previous study showed that the influencing factors for dissatisfaction after lateral PAO were the pain score and activity of daily living score of the Oxford Hip Score and the University of California, Los Angeles activity score [11]. However, no reports have identified the factors that influence patient dissatisfaction after anterior PAO. Lateral femoral cutaneous nerve (LFCN) injury is one of the known complications after anterior PAO, with a reported incidence of 48% at 1 year postoperatively [12]. Therefore, we hypothesized that LFCN injury would be a cause of patient dissatisfaction after curved periacetabular osteotomy (CPO), which is one of the anterior PAOs. This study was performed to evaluate clinical outcomes based on PROMs using the Harris Hip Score (HHS) [13], the Japanese Orthopaedic Association Hip Disease Evaluation Questionnaire (JHEQ) [14] and the Medical Outcomes Study 36-Item Short-Form Health Survey (SF-36) [15] and to analyze the factors that impact patient dissatisfaction at 1 year after CPO.

## METHODS

### Patient selection

This study was approved by our institutional review board (approval no. U22-02-015). We retrospectively evaluated 119 hips of 110 consecutive patients who underwent PAO for the treatment of symptomatic dysplastic hips from March 2016 to June 2020. The exclusion criteria were as follows: (i) the second operated side of patients who underwent bilateral CPO within the research period; (ii) the first operated side of patients who underwent contralateral CPO within 1 year of the initial PAO; (iii) a history of trauma or surgery (i.e. hip arthroscopy) of the hip on the operative side; (iv) cerebral palsy and (v) loss to follow-up.

At our institution, CPO [16] has been performed since 1995 as a modified Bernese PAO developed by Ganz *et al*. [2]. Indications for CPO include acetabular dysplasia with symptoms (e.g. pain) that are tolerable but uncomfortable and have caused some limitations in daily activities for >5 months; a lateral center-edge angle (LCEA) [17] of <25° on anteroposterior (AP) radiographs in the supine position; improvement in joint congruency on an AP pelvic radiograph with the hip in abduction; closed triradiate cartilage and age of <65 years at the time of surgery. All surgical procedures were performed by three senior orthopedic surgeons at a single institution.

### Surgical technique and postoperative rehabilitation

**Fig. 1.**
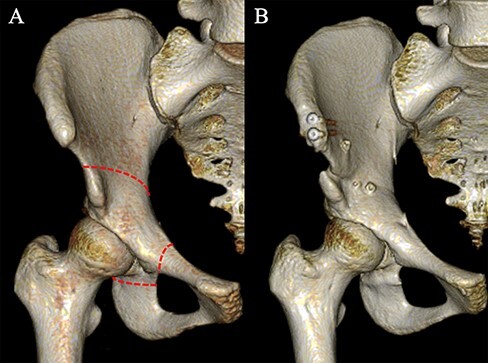
Preoperative and postoperative three-dimensional CT of the pelvis in the AP view. (A) Preoperative pelvis. The graphic shows the osteotomy line of CPO (dotted line). (B) Postoperative pelvis. The acetabular fragment was rotated laterally (to make the weight-bearing area horizontal) and fixed with three poly-l-lactic acid screws.

An anterior incision was made from the anterior superior iliac spine (ASIS) and extended distally for ∼8 cm along the tensor fasciae latae muscle. The ASIS was osteotomized with the inguinal ligament, and the remaining sartorius muscle was attached and retracted medially. The iliopsoas muscle was partially detached around the inner table of the pelvis and retracted medially with the femoral artery, vein and nerve. A C-shaped osteotomy line was started proximal to the anterior inferior iliac spine and ended at the distal part of the quadrilateral surface. The ischium was osteotomized using image intensifier guidance. Osteotomy of the superior ramus of the pubis was performed just medial to the iliopubic eminence. The acetabular fragment was rotated laterally to make the weight-bearing area horizontal and fixed with three poly-l-lactic acid screws without an iliac bone graft. The osteotomized ASIS was then repositioned and fixed with two titanium cannulated cancellous screws (Fig. 1).

Active motion exercises were started on the first postoperative day. Partial weight-bearing was started at 10 kg on the second postoperative day for patients treated before June 2017. Partial weight-bearing was started at 20 kg in the second postoperative week for patients treated after June 2017. Weight-bearing was increased by 10 kg every 2 weeks in both periods. This rehabilitation protocol was revised during the study period to prevent postoperative fracture at the posterior column. Full weight-bearing was allowed at 2–3 months postoperatively, and a return to heavy work and sports activities was allowed at 6 months postoperatively.

### Data collection

A clinical assessment based on the HHS, the JHEQ and the SF-36 was conducted preoperatively and at 1 year after PAO. The HHS (except the range of motion and deformation) had a total of 91 points, including 44 points for pain items and 47 points for function items. The JHEQ evaluates three categories using an 84-point scale: pain, motion and mental health with 7 items each, for a total of 21 items. The JHEQ also includes a visual analog scale (VAS) score for dissatisfaction and pain. Patients were divided into two groups: satisfied group and unsatisfied group according to 85th percentile cut-off on the postoperative VAS score for dissatisfaction. The SF-36 is a set of generic, coherent and easily administered measures of the quality of life. We used summary scores including the physical component score (PCS), mental component score and role component score [18]. Furthermore, LFCN injury was evaluated at 1 year postoperatively by two orthopedic surgeons. LFCN injury was defined as the presence of hypoesthesia (numbness and dull sensation) over the lateral aspect of the thigh or abnormal sensation (e.g. paresthesia, dysesthesia, hyperesthesia and causalgia).

AP radiographs were assessed at preoperatively and 1 year postoperatively. AP radiographs were taken in the supine position and standardized by a tube-to-film distance of 120 cm, and the tube was oriented perpendicularly to the table. In addition, the central beam was directed toward the midpoint between the upper border of the symphysis and a horizontal line connecting the ASIS. Radiographically, acetabular dysplasia is defined as an LCEA of <25° [19]. The Tönnis grade [20] and radiographic measurements, including the LCEA, acetabular roof obliquity (ARO) [21] and head lateralization index (HLI) [22], were assessed on the AP radiographs at the same time as the clinical assessment preoperatively and postoperatively. The ratio of the HLI was calculated as the postoperative HLI divided by the preoperative HLI. The Tönnis grade was determined as follows: Grade 0, no signs of osteoarthritis; Grade 1, slight narrowing of the joint space, slight lipping at the joint margin and slight sclerosis of the femoral head or acetabulum; Grade 2, small cysts in the femoral head or acetabulum, increased narrowing of the joint space and moderate loss of sphericity of the femoral head and Grade 3, large cysts, severe narrowing or obliteration of the joint space and severe deformity of the femoral head. The Tönnis grade was evaluated independently by two orthopedic surgeons. The kappa coefficients were 0.96–0.97 (intra-observer variance) and 0.92–0.95 (inter-observer variance). Pelvic computed tomography (CT) scans were routinely performed to evaluate the osteotomy site at 1 week postoperatively and to evaluate non-union of the pubis and ilium, non-union of the ischium after posterior column fracture and ischial ramus stress fracture at 1 year postoperatively.

### Statistical analyses

The Tönnis grade was divided into categories and treated as a categorical variable. The chi-square test was used to compare categorical data such as sex, complications and the Tönnis grade. Comparisons between preoperative and postoperative values were tested using the paired-samples *t*-tests or the Wilcoxon signed-rank test. The Mann–Whitney *U* test was used to compare patients’ characteristics, clinical scores and radiographic data between the unsatisfied and satisfied groups. Pearson’s correlation coefficient was used to calculate the correlation between the postoperative VAS score for dissatisfaction and clinical evaluation findings. The minimal clinically important difference was calculated as 1/2 SD (baseline) according to the method of Norman *et al.* [23]. Multiple logistic regression analysis was performed to investigate factors impacting patient dissatisfaction after PAO; the explanatory variables were age, body mass index (BMI), postoperative Tönnis grade of ≥2, postoperative VAS score for pain, LFCN injury and radiographic complications (non-union of the pubis, non-union of the ischium after posterior column fracture and ischial ramus stress fracture). SPSS, version 20.0 (IBM Corp., Armonk, NY, USA), was used for statistical analysis. A *P*-value of <0.05 was considered statistically significant.

## RESULTS

**Fig. 2.**
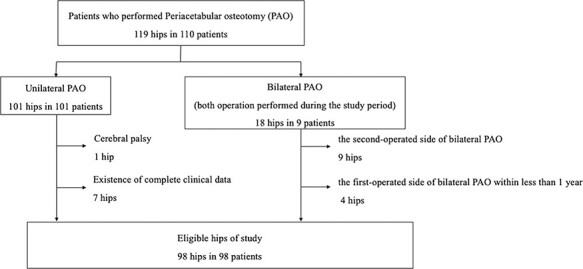
The flow chart of patient eligibility.

For nine patients who underwent bilateral CPO within the research period, the second operated side (nine hips) was excluded. Furthermore, for four patients who underwent contralateral CPO within 1 year of the initial CPO, the first operated side (four hips) was excluded. One patient with cerebral palsy was excluded because this patient did not have classic dysplasia. Among the remaining 105 hips, 7 hips of 7 patients were lost to follow-up; 2 of whom moved to another prefecture, 1 of whom studied abroad, 1 of whom was admitted to another hospital for another disease and 3 of whom did not visit our institution during the study period. Finally, 98 hips of 98 patients were included. The follow-up rate was 93.3% (98/105) (Fig. 2). No patients had a history of trauma or surgery of the hip on the operative side. The mean age of the patients at the time of surgery was 36.7 years (range, 13–60 years), and 6 male and 92 female patients were included. The average BMI was 23.0 kg/m^2^ (range, 16.4–35.6 kg/m^2^), and the mean follow-up period was 12.4 months (range, 12–17 months). All patients complained of hip pain at rest and during activity. According to the Tönnis grade, 79 hips were classified as Grade 1, 16 hips were classified as Grade 2 and 3 hips were classified as Grade 3. The original and revised rehabilitation protocols were used on 28 and 70 hips, respectively.

The preoperative and postoperative radiographic data and clinical evaluations are presented in Table I. The mean LCEA, ARO and HLI improved significantly. The mean preoperative JHEQ score was 41.2 points, which improved to a mean of 62.6 points postoperatively (*P* < 0.001). The PCS and role component score of the SF-36 significantly improved from 34.4 to 45.6 points (*P* < 0.001) and from 42.9 to 49.3 points (*P* < 0.001), respectively. The mean HHS improved from 65.9 to 84.3 points (*P* < 0.001). The mean postoperative VAS score for dissatisfaction was 26.5 ± 24.7 mm (range, 0–91 mm). The 85th percentile of the postoperative VAS score for dissatisfaction was 60 mm.

**Table I.** Radiographic measurements and clinical evaluations preoperatively and postoperatively

	*Preoperative*	*Postoperative*	*P-value*	*MCID*
Radiographic measurements				
LCEA (°)	12.5 ± 7.9 (−9.0 to 24.7)	37.0 ± 6.4 (19.1–57.2)	0.000	NA
ARO (°)	18.3 ± 6.5 (4.8–40.0)	−2.1 ± 6.7 (−19.1 to 14.6)	<0.001	NA
HLI	0.68 ± 0.07 (0.52–0.87)	0.61 ± 0.09 (0.40–0.82)	<0.001	NA
Clinical evaluations				
JHEQ (full score)				
Total (84)	41.2 ± 16.1 (2–84)	62.7 ± 16.0 (19–84)	<0.001	8
Pain (28)	11.0 ± 6.0 (0–28)	21.2 ± 6.2 (7–28)	<0.001	3
Motion (28)	14.8 ± 7.0 (1–28)	20.1 ± 6.5 (1–28)	<0.001	4
Mental (28)	15.4 ± 7.0 (0–28)	21.4 ± 6.2 (2–28)	<0.001	4
VAS for pain (100 mm)	64.6 ± 23.2 (0–100)	21.5 ± 20.7 (0–79)	<0.001	11
VAS for dissatisfaction (100 mm)	68.7 ± 25.0 (3–100)	26.5 ± 24.7 (0–91)	<0.001	13
SF-36				
PCS	34.4 ± 11.9 (3.4–62.0)	45.5 ± 8.8 (24.5–72.0)	<0.001	6
Mental component score	51.9 ± 10.8 (23.4–78.6)	53.0 ± 9.1 (24.8–72.0)	0.282	5
Role component score	42.9 ± 15.7 (−2.4 to 66.9)	49.3 ± 11.4 (2.5–62.9)	<0.001	8
HHS (full score)				
Total (91)	65.9 ± 11.8 (32–87)	84.3 ± 6.2 (65–91)	<0.001	6
Pain (44)	24.5 ± 8.0 (10–40)	39.2 ± 4.6 (30–44)	<0.001	4
Function (47)	41.4 ± 5.7 (21–47)	45.0 ± 3.1 (35–47)	<0.001	3

All data are expressed as mean ± standard deviation (range).

MCID, minimal clinically important difference; NA, not applicable.

No patients developed surgical site infection, non-union of the ilium osteotomy site or poly-l-lactic acid screw breakage. The incidence of posterior column fracture was 21.4% (6/28 hips) with the original protocol and 2.9% (2/70 hips) with the revised protocol. Of the six hips that demonstrated posterior column fracture with the original protocol, one case was revealed on CT 8 months after surgery due to hip pain. The other five cases with the original protocol and two cases with the revised protocol were all revealed on CT at 7 days postoperatively. The incidence of non-union of the ischium after posterior column fracture was 7.1% (7/98 hips), and the incidence of pubic non-union was 15.3% (15/98 hips). The incidence of ischial ramus stress fracture was 10.2% (10/98 hips). Delayed union was confirmed in two hips in the satisfied group at 1 year after PAO, but bone union was confirmed in the other eight hips. LFCN injury was observed in 35 of 98 (35.7%) patients at 1 year postoperatively.

There were no significant differences in any patient characteristics (age and BMI) or follow-up periods between the unsatisfied group and satisfied group (Table II). There were also no significant differences in the preoperative and postoperative radiographic measurements between the two groups (Table III). With respect to complications, there was a significant difference in LFCN injury (Table IV). There were no significant differences in any assessments in the preoperative clinical evaluation between the unsatisfied and satisfied groups; however, there were significant differences in two categories (pain and mental health) and the total JHEQ score, VAS scores for pain and dissatisfaction, the PCS of the SF-36 and the total score and pain score of the HHS postoperatively (Table V). There were strong negative correlations between the postoperative VAS score for dissatisfaction and the postoperative pain score of the JHEQ and HHS (*r* = − 0.494, *P* < 0.001, and *r* = − 0.420, *P* < 0.001, respectively) and a very strong positive correlation between the postoperative VAS score for dissatisfaction and the postoperative VAS score for pain (*r* = 0.633, *P* < 0.001) (Table VI). The multiple logistic regression analysis results showed that the postoperative VAS score for pain [odds ratio (OR), 1.064; 95% confidence interval (CI), 1.026–1.104; *P* = 0.001] and LFCN injury (OR, 6.775; 95% CI, 1.308–33.256; *P* = 0.018) were associated with postoperative dissatisfaction. The Hosmer–Lemeshow test showed adequate fitness (χ^2^ = 12.989, *P* = 0.112), and the omnibus tests of model coefficients also showed that the model made sense overall (χ^2^ = 24.146, *P* = 0.002) (Table VII).

**Table II.** Patient characteristics in unsatisfied and satisfied groups

	*Unsatisfied group (n = 15)*	*Satisfied group (n = 83)*	*P-value*
Sex, male:female (no. of hips)	1:14	5:78	0.586
Age at surgery (years)	37.7 ± 12.5 (15–54)	36.5 ± 13.4 (13–60)	0.851
BMI (kg/m^2^)	22.3 ± 2.4 (19.3–26.5)	23.1 ± 4.1 (16.4–35.6)	0.748
Follow-up (months)	12.1 ± 0.3 (12–13)	12.4 ± 1.2 (12–17)	0.288

All data are expressed as mean ± standard deviation (range).

**Table III.** Comparison of radiographic measurements preoperatively and postoperatively in unsatisfied and satisfied groups

	*Unsatisfied group (n = 15)*	*Satisfied group (n = 83)*	*P-value*
Preoperative evaluations			
Tönnis grade, Grade 0:1:2:3	12:2:1:0	67:14:2:0	0.654
LCEA (°)	10.7 ± 11.0 (−9.0 to 23.0)	12.8 ± 7.2 (−6.9 to 24.7)	0.745
ARO (°)	20.0 ± 8.5 (4.8–40.0)	18.1 ± 6.1 (5.7–31.4)	0.300
HLI (°)	0.67 ± 0.08 (0.52–0.81)	0.68 ± 0.07 (0.54–0.87)	0.801
Postoperative evaluations			
Tönnis grade, Grade 0:1:2:3	11:3:1:0	62:17:4:0	0.956
LCEA (°)	35.9 ± 6.7 (24.0–48.4)	37.2 ± 6.4 (19.1–57.2)	0.482
ARO (°)	0.2 ± 7.4 (−15.5 to 12.6)	−2.6 ± 6.6 (−19.1 to 14.6)	0.150
HLI (°)	0.62 ± 0.11 (0.46–0.80)	0.61 ± 0.09 (0.40–0.82)	0.863
Ratio of HLI	0.92 ± 0.09 (0.71–1.04)	0.90 ± 0.10 (0.61–1.10)	0.587

All data are expressed as mean ± standard deviation (range).

**Table IV.** Comparison of complications in unsatisfied and satisfied groups

	*Unsatisfied group (n = 15)*	*Satisfied group (n = 83)*	*P-value*
Non-union of the ischium after posterior column fracture	1 (6.7%)	6 (7.2%)	0.938
Non-union of the pubis	1 (6.7%)	14 (17.9%)	0.313
Ischial ramus stress fracture	3 (20.0%)	7 (8.4%)	0.173
LFCN injury	9 (60.0%)	26 (31.3%)	0.033

All data are expressed as *n* (%).

**Table V.** JHEQ score, HHS and SF-36 score preoperatively and postoperatively in unsatisfied and satisfied groups

	*Unsatisfied group (n = 15)*	*Satisfied group (n = 83)*	*P-value*
Preoperative			
JHEQ (full score)			
Total (84)	39.7 ± 7.3 (25–51)	41.4 ± 17.2 (2–84)	0.527
Pain (28)	10.5 ± 3.6 (4–16)	11.1 ± 6.3 (0–28)	0.839
Motion (28)	13.2 ± 3.3 (7–18)	15.1 ± 7.4 (1–28)	0.421
Mental (28)	16.1 ± 6.0 (5–28)	15.3 ± 7.3 (1–28)	0.534
VAS for pain (100 mm)	66.8 ± 21.4 (27–100)	64.3 ± 23.6 (1–100)	0.906
VAS for dissatisfaction (100 mm)	70.1 ± 22.8 (29–100)	68.4 ± 25.4 (3–100)	0.976
SF-36			
PCS	32.3 ± 6.8 (22.7–45.8)	34.8 ± 12.6 (3.4–62.0)	0.264
Mental component score	54.4 ± 8.5 (43.7–76.9)	51.4 ± 11.2 (23.4–78.6)	0.331
Role component score	44.1 ± 14.9 (13.3–59.7)	42.6 ± 15.9 (−2.4 to 66.9)	0.697
HHS (full score)			
Total (91)	61.9 ± 10.4 (45–77)	66.6 ± 11.9 (32–87)	0.095
Pain (44)	20.7 ± 8.0 (10–30)	25.2 ± 7.9 (10–40)	0.059
Function (47)	41.2 ± 3.2 (35–47)	41.4 ± 6.1 (21–47)	0.258
Postoperative			
JHEQ (full score)			
Total (84)	53.1 ± 13.3 (35–71)	64.4 ± 15.9 (19–84)	0.011
Pain (28)	16.9 ± 7.5 (7–28)	22.0 ± 5.6 (7–28)	0.012
Motion (28)	17.7 ± 4.9 (7–28)	20.5 ± 6.7 (1–28)	0.100
Mental (28)	18.5 ± 5.2 (9–26)	21.9 ± 6.2 (2–28)	0.017
VAS for pain (100 mm)	40.1 ± 25.5 (1–79)	18.2 ± 17.9 (0–70)	0.002
VAS for dissatisfaction (100 mm)	71.5 ± 9.0 (60–91)	18.3 ± 16.4 (0–59)	<0.001
SF-36			
PCS	41.1 ± 7.7 (30.9–58.3)	46.4 ± 8.8 (25.4–72.0)	0.031
Mental component score	54.1 ± 7.7 (34.8–63.3)	52.7 ± 9.4 (24.8–72.0)	0.592
Role component score	50.2 ± 9.7 (25.7–60.5)	49.2 ± 11.7 (2.5–62.9)	0.898
HHS (full score)			
Total (91)	80.7 ± 7.1 (69–87)	84.9 ± 5.8 (65–91)	0.017
Pain (44)	36.9 ± 5.2 (30–44)	39.7 ± 4.4 (30–44)	0.028
Function (47)	43.8 ± 3.5 (36–47)	45.3 ± 2.9 (35–47)	0.065

All data are expressed as mean ± standard deviation (range).

**Table VI.** Correlations between postoperative VAS score for dissatisfaction and postoperative clinical evaluation results

	*Correlation coefficient, r*	*P-value*
JHEQ		
Total	−0.441	<0.001
VAS for pain	0.633	<0.001
Pain	−0.494	<0.001
Motion	−0.275	0.006
Mental	−0.359	<0.001
SF-36		
PCS	−0.333	<0.001
Mental component score	−0.087	0.342
Role component score	−0.420	<0.001
HHS		
Total	−0.386	<0.001
Pain	−0.420	<0.001
Function	−0.154	0.130

**Table VII.** Multiple logistic regression analysis of factors associated with postoperative dissatisfaction

	*OR*	*95% CI (lower limit)*	*95% CI (upper limit)*	*P-value*
Age	0.982	0.924	1.043	0.551
BMI	0.892	0.714	1.113	0.312
Postoperative VAS for pain	1.064	1.026	1.104	0.001
Postoperative Tönnis grade ≥2	15.631	0.593	412.330	0.100
Non-union of the ischium after posterior column fracture	1.788	0.104	30.803	0.689
Non-union of the pubis	4.512	0.033	4.894	0.475
Ischial ramus stress fractures	4.251	0.386	46.773	0.237
LFCN injury	6.775	1.380	33.256	0.018

## DISCUSSION

This study based on PROMs showed improvements in pain, function, mental health and satisfaction. This result is similar to the results of other studies based on PROMs [7, 9, 10]. Both the JHEQ score and the HHS reflect patients’ postoperative satisfaction. A study comparing patient satisfaction with PAO and total hip arthroplasty (THA) reported that PAO was more satisfactory than THA for early-grade osteoarthritis but that THA was more satisfactory than PAO as the osteoarthritis grade progressed [11]. CPO is one of the most satisfying procedures for patients with acetabular dysplasia, but to achieve a high level of satisfaction, the indications must be carefully evaluated.

LFCN injury and the postoperative VAS score for pain independently impacted postoperative dissatisfaction. Previous studies have shown that the incidence of LFCN injury after anterior-approach PAO was 65–74% at ∼2 weeks postoperatively, 48% at 1 year postoperatively and 30% at 7.4 years postoperatively [12, 24, 25]. Although the symptoms of LFCN injury tend to decrease with time, avoiding intraoperative LFCN injury is important to achieve high patient satisfaction after surgery and is one of the greatest challenges of anterior-approach PAO.

In this study, we were able to investigate the degree of postoperative pain using VAS scores, but we were not able to investigate the cause of pain in detail. The causes of pain after PAO include non-union of the ischium after posterior column fracture, non-union of the osteotomy site, stress fracture, progression of osteoarthritis, femoroacetabular impingement and muscle–tendon pain around the hip joint [26–28]. The number of posterior column fractures decreased after the protocol revision; thus, one of the causes of posterior column fracture may have been early postoperative loading. However, posterior column fractures did continue to occur after the protocol revision, suggesting the presence of unintentional intraoperative surgical injuries. When osteotomizing the ischium using the chisel, the image intensifier guide should be used and the blade should be oriented carefully. If a posterior column fracture occurs, it is advisable to delay the start of loading. It has been previously reported that a gap of >5.1 mm at the pubis osteotomy site is a risk factor for pubic non-union [29] and that pubic non-union is an inducer of ischial ramus stress fracture [30]. These issues may be avoidable with surgical techniques, and pain associated with surgery should be avoided as much as possible.

The present study has several limitations that should be noted. First, the level of evidence in this retrospective, single-center study is insufficient compared with prospective, multicenter studies. Second, the number of male patients was small because of the nature of the disease. Third, this study was designed based on a 1-year follow-up period, as a 1-year evaluation after surgery may be meaningful even for performing the longer-period follow-up study. The factors that impact patient satisfaction may change over time, and further research on medium- to-long-term outcomes would be useful. Finally, we did not investigate whether any treatment was given for postoperative pain. However, none of the patients underwent reoperation for postoperative pain within 1 year after CPO.

## CONCLUSION

This study based on PROMs showed that CPO was a satisfactory surgery that improved pain, motion and mental health. In contrast, LFCN injury and the postoperative VAS score for pain independently impacted postoperative dissatisfaction. These results suggest the importance of avoiding LFCN injury, posterior column fracture and pubic non-union, which cause postoperative pain, to improve patient satisfaction.

## Data Availability

The data underlying this article will be shared on reasonable request to the corresponding author.
